# Minimal residual disease detection in Tunisian B-acute lymphoblastic leukemia based on immunoglobulin gene rearrangements

**DOI:** 10.1590/1414-431X20165426

**Published:** 2017-01-16

**Authors:** S. Besbes, W.S. Hamadou, M.L. Boulland, Y.B. Youssef, B. Achour, H. Regaieg, A. Khelif, T. Fest, Z. Soua

**Affiliations:** 1 Research Unit 14 ES 19, Department of Biochemistry, Faculty of Medicine, University of Sousse, Sousse, Tunisia; 2 Biological Hematology Department, Centre Hospitalier Universitaire Pontchaillou, Rennes, France; 3 Clinical Hematology Department, Hospital F. Hached, Sousse, Tunisia

**Keywords:** *IGH* gene, *IGK* gene, B-ALL minimal residual disease (MRD), RQ-PCR

## Abstract

*IGH* gene rearrangement and *IGK-Kde* gene deletion can be used as molecular markers for the assessment of B lineage acute lymphoblastic leukemia (B-ALL). Minimal residual disease detected based on those markers is currently the most reliable prognosis factor in B-ALL. The aim of this study was to use clonal *IGH/IGK-Kde* gene rearrangements to confirm B-ALL diagnosis and to evaluate the treatment outcome of Tunisian leukemic patients by monitoring the minimal residual disease (MRD) after induction chemotherapy. Seventeen consecutive newly diagnosed B-ALL patients were investigated by multiplex PCR assay and real time quantitative PCR according to BIOMED 2 conditions. The vast majority of clonal VH-JH rearrangements included VH3 gene. For IGK deletion, clonal VK1f/6-Kde recombinations were mainly identified. These rearrangements were quantified to follow-up seven B-ALL after induction using patient-specific ASO. Four patients had an undetectable level of MRD with a sensitivity of up to 10^-5^. This molecular approach allowed identification of prognosis risk group and adequate therapeutic decision. The *IGK-Kde* and *IGH* gene rearrangements might be used for diagnosis and MRD monitoring of B-ALL, introduced for the first time in Tunisian laboratories.

## Introduction

Acute lymphoblastic leukemia (ALL) is a malignant disorder of lymphoid progenitor cells. It is thought to originate from various important genetic lesions in blood-progenitor cells that are committed to differentiate in T or B-cell pathway. ALL represents 45.1% of all hematological malignancies according to the last Tunisian Cancer Register with a standard incidence of 2.25. B-ALL is the most common with a frequency of 71%, of which 78% are children and 50% are adults, while T-ALL represents 29%, which is higher than in the European population (1). Tunisian B-ALL patients show a high rate of relapse with a short complete remission.

The level of residual leukemic cells is currently the most reliable prognosis factor in B-ALL. Assessment of minimal residual disease (MRD) performed after induction allows the stratification of patients into risk groups and the adjustment of treatment strategy (2). During the last decade, several molecular markers and techniques have been developed for the MRD quantification and evaluation of the clonal leukemic population, exhibiting higher sensitivity than the morphological approach. For B-leukemias, immunoglobulin (IG) gene rearrangement monitoring is commonly used (3,4).

During the V_H_D_H_J_H_ recombination, the high sequence homology between the seven VH subgroups (IGHV1–IGHV7) is useful and favorable for *IGH* gene amplification with consensus primers (5–8). The IGK locus is deleted through the kappa deleting element (Kde) and two different types of Kde recombination can inactivate non-functional IGK rearrangements: Kde rearranges to the intron RSS resulting in the deletion of the IGKC gene and the maintenance of the IGKV-J junction (9,10). Therefore, it is possible to detect both IGKV-J and intron-Kde rearrangements on the same allele (11,12). The Kde segment can also rearrange to one of the *VK* genes with the subsequent loss of the IGKV-J junction.

For each IGH or IGK recombination, insertion of nucleotides occurs at the VDJ or VK-Kde/RSS-Kde junctions respectively leading to the N-region, which constitutes a molecular sequence tag for each lymphocyte (13–16). Since almost 90% of B-ALL rearrange the IGH locus and 50% delete the IGK-Kde locus, these recombined loci are used as patient-specific "fingerprint-like" markers of residual leukemic cells (17) for the assessment of MRD in leukemia. Molecular MRD monitoring is widely performed by real time quantitative PCR. The consortium of BIOMED-2 (17) and more recently "EuroClonality" have proposed a panel of novel PCR-based methods allowing the detection of IG rearrangements in order to support the accurate and reliable diagnosis and follow-up of hematological malignancies, especially in lymphoid lineage.

The diagnosis and MRD monitoring of Tunisian ALL are commonly achieved through morphological and cytogenetic methods. Due to the sensitivity limits of these techniques, we have developed a molecular approach to better quantify the residual blasts even with normal cytology. This method also has a prognosis impact, as it permits to stratify patients into risk groups. The aim of this study was to use both clonal *IGH* and *IGK-Kde* gene rearrangements to confirm B-ALL diagnosis and to evaluate for the first time the treatment outcome of Tunisian patients by monitoring the MRD after induction.

## Material and Methods

### Patients

Seventeen Tunisian B-ALL patients diagnosed at the hematology department of the F. Hached Hospital of Sousse were included in this study. Informed consent was obtained from the patients or their legal guardian, as required by the Helsinki Declaration. The cohort of patients consisted of 11 males and 6 females (sex ratio: 1.83) and the median age was 6 years at the time of diagnosis (1-51 years).

### Inclusion criteria

Morphological and immunophenotypic analysis of B-ALL at diagnosis were made according to the French-American-British (FAB) and the European Group for Immunological Classification of Acute Leukemia (EGIL) criteria respectively (18,19). Cytogenetic analysis was performed using G banding technique according to the World Health Organization (WHO 2008).

### Treatment protocol

St. Jude Protocol was used for pediatric patients and Hyper CVAD for adults.

### Response criteria

Complete remission (CR) was defined as: normal bone marrow (with <5% blasts and >25% cellularity), neutrophil counts >1.5×10^9^/L, platelet count >100×10^9^/L, and extramedullary disease completely resolved.

### Material

Peripheral blood samples were obtained from the 17 patients at diagnosis with high blastosis (>75%) and from 11 patients after induction (day 35) to investigate detectable *IGH* and *IGK-Kde* rearrangements. A control population was recruited among healthy blood donors. Blood samples were obtained after the donors had given their informed consent.

### Mononuclear cells and DNA isolation

Mononuclear cells were isolated by Ficoll-Paque density centrifugation and were directly used for DNA isolation. DNA was extracted with phenol chloroform or QIAGEN DNA Blood kit (FlexiGene DNA Kit, Qiagen, USA according to the manufacturer's instructions. DNA was quantified by spectrophotometry assay and tested for quality by agarose gel electrophoresis and standard PCR amplification for the beta-globin gene.

### PCR analysis

Clonal *IGH* and *IGK-Kde* gene rearrangements were investigated by PCR amplification. For *IGH* gene rearrangements, VH family-specific primers combined with a consensus JH primer were used (17). For *IGK-Kde* rearrangements, a reverse Kde primer was used in combination with one of six VK family-specific primers or with RSS primer located at the JK-CK intron (17).

Multiplex PCR was performed according to BIOMED 2 consortium (17), and PCR products were identified by Gene Scanning using the ABI Prism 3130xL Genetic analyzer (Applied Biosystems, USA).

### Sequencing analysis

Fluorescent sequencing was performed using the Big Dye-terminator cycle sequencing kit and the ABI Prism 3130xl Genetic Analyzer (Applied Biosystems). Sequences were analyzed with sequencing analysis 5.3 software (Applied Biosystems), identified using V-quest IMGT (http://www.imgt.org/IMGT_vquest/vquest?livret=0&Option=humanIg) and confirmed with Ig-BLAST sequence similarity searching tool (http://www.ncbi.nlm.nih.gov/igblast/).

### ASO primers design

All used TaqMan probes and consensus primers have been previously described (8,20). The allele-specific oligonucleotide (ASO) forward primers were designed using the Primer 3 Software (http://primer3.wi.mit.edu/). The ASO primer was complementary to the IGH/IGK-Kde junctional region and was designed for each patient and tested for sensitivity and specificity.

### RQ-PCR analysis

The MRD quantification was performed by real time quantitative PCR (RQ-PCR) using patient-specific *IGH/IGK-Kde* gene rearrangement primers, in combination with a set of different germline TaqMan probes and reverse germline primers according to the BIOMED 2 conditions (8,17,20,21). MRD levels were stated as the proportion of leukemic cells in all nucleated cells in the sample. The amplification efficiency and the RQ-PCR target sensitivity were tested by serially diluting (from 10^−1^ down to 10^−5^) the diagnosis DNA in control DNA from a pool of 8 healthy donors. Diagnosis and follow-up samples were analyzed in duplicate and triplicate, respectively. The albumin gene was included in RQ-PCR analysis for quality DNA adjustment.

## Results

### Biological characteristics of B-ALL

In the 17 B-ALL patients analyzed at diagnosis, the myelogram identified more than 20% of blasts. Immunophenotyping showed low intensity of the CD45, allowing the gate of the blasts expressing the HLA-DR and CD34. The B-ALL were CD79a^+^, CD22^+^ and CD19^+^ and subdivided according to the EGIL classification. Cytogenetic analysis showed normal karyotype in 8 cases (Table 1). Six B-ALL patients presented chromosomal aberrations: 3 hyperdiploidies, 1 translocation t(1;19)(q23;p13), 1 structural anomaly of chromosome 21 and a marker chromosome.

**Table:**
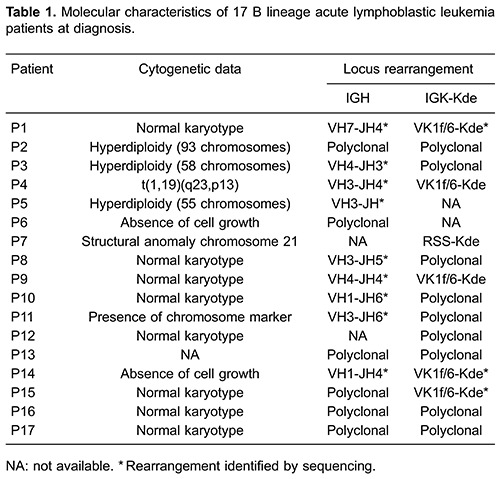

All patients showed corticotherapy sensitivity at day 8 and achieved clinical remission after the first course of chemotherapy (Table 2).

**Table:**
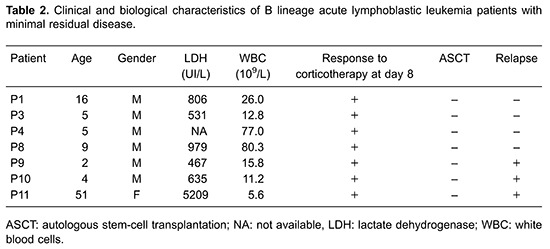

### IGH and IGK-Kde rearrangements analysis at diagnosis

**Figure 1:**
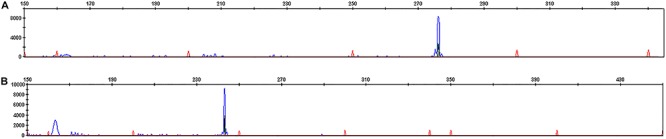
GeneScan of IG rearrangement in a B lineage acute lymphoblastic leukemia patient (P14). *A*, Monoclonal VH-JH rearrangement; *B*, monoclonal VK-Kde rearrangement.

The multiplex PCR allowed the amplification of 15 clonal IGH and IGK-Kde rearrangements (Table 1). To study IGH rearrangement, we used 7 primers (VH1 to VH7) combination in the multiplex PCR assay. We amplified in 9 patients one pic at the expected 250–295 pb size corresponding to VH-JH rearrangement (Figure 1A). The vast majority of rearrangements included *VH3* gene, followed by VH1, VH4 and VH7. In each patient we have also determined the JH segment (JH3, 4, 5, 6) by direct sequencing.

For IGK-Kde rearrangement, we used 7 primers (VK1f/6 to VK7 and intron RSS) combination in the multiplex PCR assay. We amplified in 6 patients one pic at the expected 120–300 pb size corresponding to VK-Kde or RSS-Kde rearrangement (Figure 1B). Five patients showed VK1f/6-Kde rearrangement and only 1 presented the intron RSS-Kde rearrangement. Concomitant rearrangements of both IGH and IGK loci were observed in 4 cases (Table 1).

### Sequencing analysis

Among the 15 amplified IG rearrangements, only 8 IGH and 3 IGK-Kde rearrangements were successfully sequenced. Negative results of sequencing or insufficient DNA specimens did not allow data analysis. The sequenced N-region confirmed the occurrence of VH-JH and VK-Kde rearrangements as determined by multiplex PCR (Table 3). Unfortunately, only the sequence of RSS-Kde rearrangement was not possible. The number of nucleotides in VH-JH or VK-Kde junctional region was identified (Table 3). The overall mean number of insertion was 6.9 (range: 0–24) and the overall mean number of deletions was 10.6 (range: 0–26). The N-region allowed the ASO construction in 8 IGH and only 2 IGK-Kde junctional regions.

**Table:**
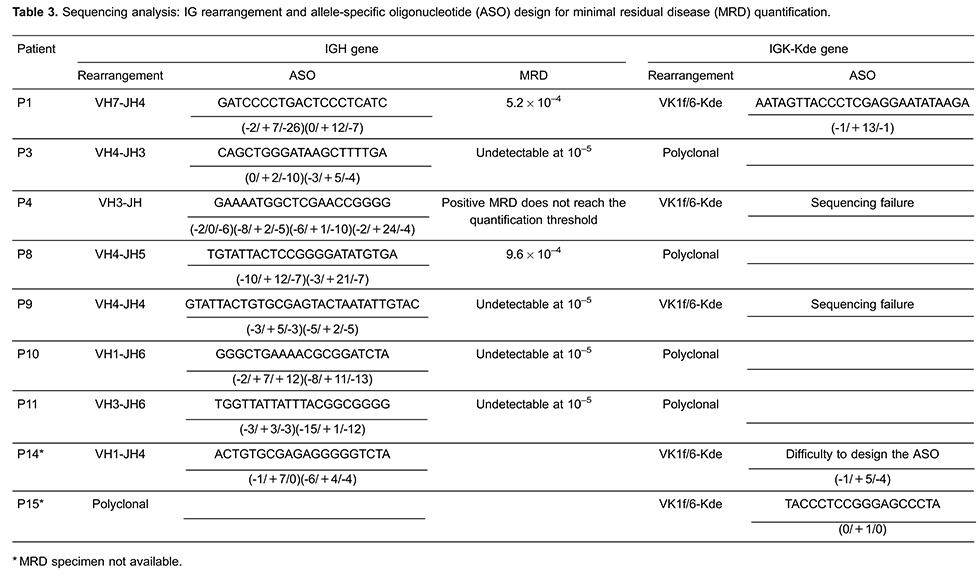

### MRD evaluation

Cytomorphological analysis was performed after induction phase (day 35) in 11 patients (P1 to P11) among the 17 cases. Five patients were in cytological remission with less than 5% of blasts, whereas 4 patients (P1, P3, P9, P10) showed no cytological remission. In 2 cases (P2, P4) results were inconclusive due to poor preparation of smears or low concentration of sample.

As IGH and IGK-Kde rearrangements are highly stable PCR targets with relatively large junctional regions, we have applied in this study these rearrangements for MRD monitoring in B-ALL cases by RQ-PCR.

**Figure 2:**
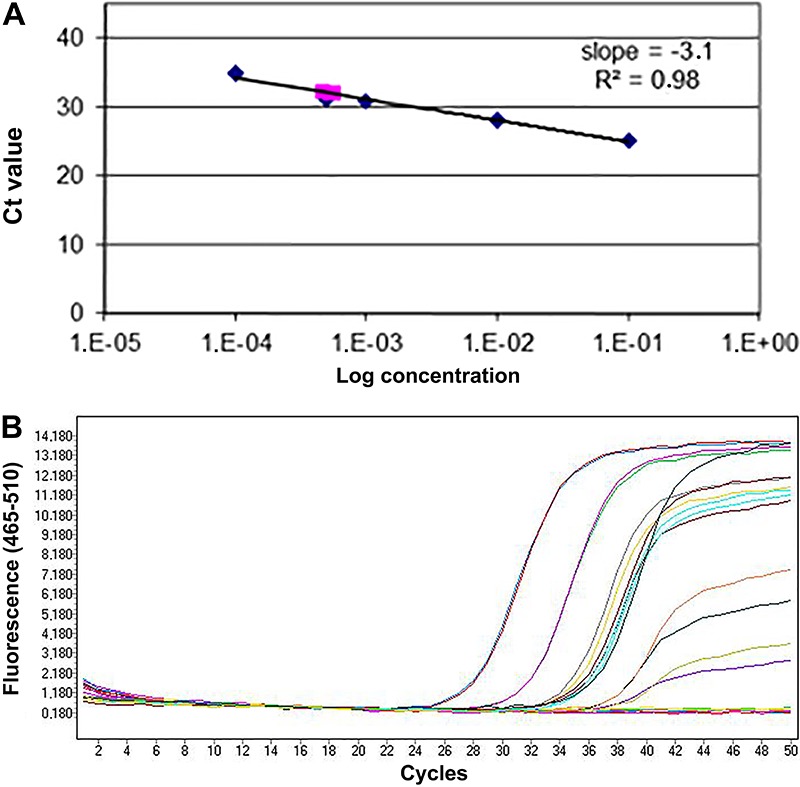
Minimal residual disease monitoring of a B lineage acute lymphoblastic leukemia (P1) using VH-JH rearrangement. *A*, standard curve; *B*, amplification curves.

**Figure 3:**
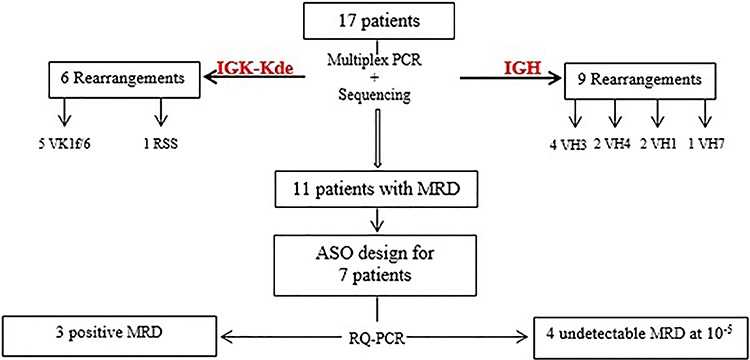
Consort diagram of patients. MRD: minimal residual disease; ASO: allele-specific oligonucleotide.

To provide a quantification assay, standard curves were drawn with serial dilution of the diagnosis DNA into control DNA. The RQ-PCR assay was performed with each patient DNA and its quality was compared to amplification results of housekeeping albumin gene. Data were reported using a common threshold of 1. ASO primers were tested for their sensitivity in 7 IGH N-regions and only in 1 IGK-Kde N-region due to unavailable MRD points (Table 3). For all B-ALL cases, standard curves exhibited a slope between –3.1 and –3.9 and a correlation coefficient of at least 0.98 for precise quantification (Figure 2A). For IGH-ASO primers, the sensitivity reached the threshold of 10^−5^ and the amplification curves allowed the evaluation of the MRD level at Ct value (Figure 2B). Four patients had an undetectable level of MRD (P3, P9, P10, P11) (Figure 3). Two patients (P1, P8) showed residual blasts with a rate of 5.2×10^−4^ and 9.6×10^–4^, respectively. In 1 case (P4), the residual blasts were in an overall quantification area (Table 3). For IGK-Kde ASO primer, the sensitivity reached the threshold of 10^−5^ and 1 patient (P1) had a high MRD level of 6×10^−4^ (Table 3).

## Discussion

In the B-ALL group, remission was obtained in 98% of cases, whereas only 85% of Tunisian patients were in remission within weeks after starting treatment (22). The therapeutic failure after induction and the mortality rate remained high (5 and 6%, respectively). Several prognostic factors must be taken into account to identify these high risk patients: initial features at presentation of the disease (age, leukocyte count, immunophenotype, chromosomal translocations); response to chemotherapy and measurement of MRD during the first period of therapy. The threshold for predicting relapse depends on the time-point of detection: a high leukemic residual cells after induction increase the risk, and patients might benefit from different treatment strategies or bone marrow transplantation. The absence of residual disease monitoring in Tunisian patients and the lack of precise classification of B-ALL into risk groups may explain the high risk of relapse, which encouraged us to introduce a molecular approach to improve prognosis and monitoring of B-ALL.

IGH rearrangements and IGK-Kde deletions can be used for diagnosis and follow-up of B-lineage leukemias since they are identified in nearly 90 and 50% of B-ALL, respectively (3,23-25). MRD assessment during the treatment is a powerful prognosis indicator in B-ALL and requires high sensitivity of the PCR-based techniques for best clinical implementation (26).

In this study, we introduced *IGH* and *IGK-Kde* genes rearrangement as an independent diagnostic and prognostic factors in Tunisians with B lineage leukemias. We analyzed 17 diagnosed patients and investigated for the first time the molecular MRD quantification of leukemic patients in order to improve their follow-up. The IGH and IGK-Kde multiplex PCR assays were performed using BIOMED 2 conditions. The presence of clonal proliferation was determined in nine B-ALL through IGH rearrangements. During VHJH rearrangement, the usage of *VH* gene was similar to published data: 44% VH3 (*vs* 30–50%), 22% VH4 (*vs* 20–30%), 22% VH1 (*vs* 10–20%) and 11% VH7 (27). VH6 gene was not rearranged here although it is frequently used in B-ALL (17). We found the clonal IGK deletion in six patients with high frequency of Vκ1f/6 (83% *vs* 68–100%) and lower of intron RSS (16% *vs* 0–31%) (3,17,28). Four patients had simultaneous rearranged IGH and IGK-Kde loci, which represent two powerful and helpful tools for determining the clonality of the vast majority of B-leukemias. These rearrangements were identified in one multiplex PCR assay, which is highly specific for detection and identification of the most common IGH rearrangements and Kde deletional rearrangements. The PCR-based techniques offer a good alternative for B lineage leukemias diagnosis, established in our clinical department by morphological and immunophenotypic methods (24).

Since IGH/IGK-Kde rearrangements are stable during the disease follow-up (17), the junctional N-region allows patient-specific ASO primers design. Sequencing analysis revealed a mean insertion of 6.9 nucleotides (*vs* 3.0 in published data) (29) and a mean deletion of 10.6 nucleotides (*vs* 10.4 in published data) (29) at the junctional regions of rearrangements. The N-region is a potential molecular target for MRD monitoring in each single patient. The MRD assessment allows blast clearance evaluation and treatment adaptation. After induction, blast clearance is a prognosis factor if complete remission is achieved early (30). It is usually correlated with the cytological appearance of bone marrow as well as the persistence of circulating blast cells, which generally reflects a poor prognosis (30). In this study, 50% of B-ALL patients showed blast clearance at day 35. This rate is less than the 60–70% published yield and is in favor of a poor blast clearance.

Eleven of the 17 patients had available samples at day 35. Response to B-ALL treatment depends on many factors including: the clinical and biological characteristics of the disease, chemotherapy used, the patient's ability to metabolize anti-cancer treatment and the prognosis value of cytological aspect of the bone marrow at day 8. In our series, the blast clearance after induction was often correlated with the cytological appearance of bone marrow in day 8, which is a good prognosis factor. Six patients were monitored both by cytomorphology and IG investigation, which allowed their comparison. Two patients had concordant results using these two approaches. P1 showed a 5% of blastosis and a positive and similar MRD level (>10^−4^) as deduced by two IGH and IGK-Kde rearrangements, whereas P11 exhibited both a cytological remission (<5%) and an undetectable MRD level. Four patients showed discordant results. Three of them (P3, P9, P10) having a blastosis greater than 5% at day 35 but an undetectable MRD level (below 10^−4^) and 1 case (P8) presenting complete remission after induction with a <5% cytological level of blasts but a positive molecular MRD level (>10^−4^). These discrepancies might be due to the difficulty of cytological techniques to distinguish between newly regenerated cells and leukemic blasts, and to the method sensitivity which does not exceed 10^−2^. We observed a fairly high MRD level, which may be used to classify these patients as a high-risk group, who will benefit from therapy intensification. As these patients continued to receive maintenance therapy they did not relapse and were chemotherapy-sensitive. Patients who relapsed may exhibit resistance to chemotherapy. Additional time points are needed in order to follow the kinetic of MRD evolution for better relapse prediction. Otherwise, we can correlate these molecular data to disease-free-survival if we have sufficient feedback (more than 3 years). van Dongen et al. (31) have demonstrated that MRD is correlated with 3 to 5% of relapse at 3 years in patients with a negative MRD (<10^−4^) and 39–46% of relapse in 3 years in the positive MRD group (>10^−4^). More time-points during treatment are needed to better monitor MRD in our patients and forecast relapse (Table 2).

Cytogenetic aberrations correlate strongly with clinical outcome and can identify leukemia subgroups with different responses to therapy (32). In 1 patient (P4), both positive MRD at day 35 and the presence of the translocation t(1;19)(q23;p13) according to E2A-PBX fusion gene, are in favor of a poor prognosis. Hence molecular and cytogenetic data allow a better stratification of patients.

The most obvious application of the MRD test, is its use in measuring early treatment response and identifying patients who achieve morphologic remission but still harbor high MRD level.

The association of clinical features including sex, white blood cell, translocation, etc and positive MRD in patients P1, P4 and P8 allowed their classification in the high risk group despite normal cytological data. Molecular results were in favor of poor prognosis. In 1 patient (P3) showing a negative MRD, we did not change the therapeutic protocol despite the worst cytological and clinical features. This patient did not relapse. This highlights the importance of MRD as independent prognostic factor.

In conclusion, we have successfully introduced and applied molecular IGH/IGK-Kde rearrangements to confirm diagnosis and assess for the first time MRD evaluation of B-ALL, which have been investigated only with cytological and immunophenotypic analysis. We presented the MRD evaluation feasibility, which will be used in a larger cohort study and benefit other patients from a better adapted therapy. This molecular approach will be introduced in clinical routine in Tunisian hematological laboratories. The MRD is an important predictive factor in B-ALL patients leading to individualized treatment. This may avoid the unnecessary consolidations in some low-risk patients while alerting for the need of transplantation in others.
